# Cross-cultural validation of the Bengali version KIDSCREEN-27 quality of life questionnaire

**DOI:** 10.1186/s12887-018-1373-7

**Published:** 2019-01-15

**Authors:** Rosalie Power, Rahena Akhter, Mohammad Muhit, Sabrina Wadud, Eamin Heanoy, Tasneem Karim, Nadia Badawi, Gulam Khandaker

**Affiliations:** 1 Discipline of Child and Adolescent Health, Faculty of Medicine and Health, University of Sydney, Sydney, New South Wales Australia; 2 Asian Institute of Disability and Development (AIDD), University of South Asia, Dhaka, Bangladesh; 3 Faculty of Dentistry, University of Sydney, Westmead, New South Wales Australia; 4 CSF Global, Bangladesh, Dhaka, Bangladesh; 5 Cerebral Palsy Alliance Research Institute, University of Sydney, Sydney, New South Wales Australia; 6 Public Health Unit, Central Queensland Hospital and Health Service, Rockhampton, Queensland, Australia; 7 The Children’s Hospital at Westmead (Clinical School), Cnr Hawkesbury Rd and Hainsworth St, Locked Bag 4001, Westmead, NSW 2145 Australia

**Keywords:** KIDSCREEN-27, Health-related quality of life (HRQoL), Cerebral palsy (CP), Disability, Adolescent, Teenager, Bangladesh, Low and middle-income country (LMIC), Psychometric properties, Validation

## Abstract

**Background:**

Measuring the health-related quality of life (HRQoL) of adolescents, including those with cerebral palsy (CP) (the major cause of childhood physical disability worldwide) in Bangladesh is pertinent although there is a dearth of validated instruments for assessing this concept. For application in a case-control study comparing HRQoL between adolescents with CP and peers without disability in Bangladesh (a typical low- and middle-income country) we cross-culturally translated and psychometrically tested KIDSCREEN-27.

**Methods:**

KIDSCREEN-27 was translated to Bengali using forward and backwards translation protocol and interviewer administered to adolescents with CP and their age and sex matched peers without disability. Primary caregivers were included for proxy-report. Sociodeomgraphic characterists and clinical information were extracted from the Bangladesh Cerebral Palsy Register (BCPR) and adolescent mental health was assessed using the Bengali version Strenghts and Difficulties Questionnaire (SDQ). Feasibility, floor and ceiling effect, internal consistency, content and construct validity of KIDSCREEN-27 were tested.

**Results:**

Feasibility, floor and ceiling effect and internal consistency of KIDSCREEN-27 was good for both self- and proxy-report questionnaires; nil missing scores except ‘school environment’ (11.0% to 74.7%) which correlated to rates of non-school attendance; floor and ceiling effect ≤10.4% except ‘peers and social support’ 23.4%; Cronbach’s alpha 0.67 to 0.91. Instrument validity was strong; factor analysis reflected original instrument dimensions within one to three factors and difference in known groups was observed by CP and adolescent mental health (*p* < 0.05).

**Conclusion:**

KIDSCREEN-27 successfully translated to Bengali and both the self and proxy-report questionnaires showed good psychometric properties indicating suitability for case-control assessment of HRQoL between adolescents with CP and peers without disability in Bangladesh.

**Electronic supplementary material:**

The online version of this article (10.1186/s12887-018-1373-7) contains supplementary material, which is available to authorized users.

## Background

Health-related quality of life (HRQoL) assessment is becoming a fundamental component of public health surveillance however there is a dearth of validated instruments for assessing this concept amongst adolescents, including those with cerebral palsy (CP), and in low and middle-income countries (LMICs) such as Bangladesh [1, 2]. HRQoL, a subset of quality of life, is a subjective multidimensional concept for measuring the interaction between health status and physical, psychological, and social aspects of well-being [3]. HRQoL assessment can be used to provide understanding of burden of disease; to identify priority areas for allocation of health resources and development of public health infrastructure; policy guidance; and to deliver valid indicators of intervention outcomes (such as health service evaluation and to assess the impact of clinical interventions and treatment on quality of life) [4, 5]. Assessment of HRQoL is also useful to identify and monitor cohorts at risk of poor wellbeing such as those with CP, the major cause of childhood physical disability worldwide [6], and to assess inequality in wellbeing compared to the general population [4, 5].

Bangladesh, one of the most densely populated and under resourced countries in the world, has a large adolescent population (10 to 18 year olds) constituting approximately one fifth of the total population. Over 67% of adolescent girls are married and more than 50% will give birth before the age of 18 [7, 8]. Moreover, prevalence of CP is high. A recent population-based study estimated prevalence to be 3.4 per 1000 children [9]. Motor function tended to be severely impaired and rates of speech, visual, hearing impairments and epilepsy were all above international norms [9, 10]. Throughout Bangladesh development of public health infrastructure including disability services are at a critical stage with government focus on inclusive development guided by the Sustainability Goals and United Nations Convention on the Rights of Persons with Disability and accompanied by strong economic growth [11, 12]. Validated instruments for measuring the HRQoL of adolescents in Bangladesh are essential to guide the development of systems and service that reduce inequality and address holistic dimensions of health and wellbeing.

Adolescence, a life stage of complex physical, emotional, social and sexual development, is a pertinent time for assessment of HRQoL however to date HRQoL research in Bangladesh, including instrument validation studies, have predominately focused on adult populations and conditions of chronic illness such as type 2 diabetes, rheumatoid arthritis, obstetric fistula, kidney disease, spinal tumours and cataract. The HRQoL of adolescents with CP in Bangladesh is unknown however HRQoL assessment of children and adolescents with CP in other LMICs has indicated that wellbeing will be significantly poorer than for peers without disability [1]. Encouragingly, emerging research from high income countries (HICs) has reported that some adolescents with CP will have similar HRQoL as their peers without disability [13].

Since introduction in the 1990s measure of HRQoL has seen rapid advances and numerous instruments are available for measuring this concept, including generic population and condition-specific measures [14]. Condition-specific measures are intended to be sensitive to factors unique to their respective cohort, for example, in adolescents with CP assessment of pain and feelings about functioning are pertinent [15]. On the other hand, generic population measures can determine population norms and enable case-control comparison to identify sub-groups at risk of poor wellbeing [14].

KIDSCREEN is a generic population instrument that measures the HRQoL of children/ adolescents aged 8 to 18 years and has strong potential for adaptation to Bangladesh and among native Bengali speaking people (approximately 250 million people globally [16]). The instrument was originally developed simultaneously in 13 European counties to enable international conceptualisation of HRQoL and how it should be measured; is available in three lengths (i.e. 10, 27 or 52 items); and has both self-report and proxy-report options [17]. The instrument has currently been translated to 36 other languages and has consistently reported strong psychometric properties outperforming other generic HRQoL instruments [15]. KIDSCREEN has been tested in settings that are culturally, linguistically and religiously diverse to each other [18, 19] however has not yet been used in South Asia and of the 36 language translations, only two are from countries which are LMICs and in which Islam is the dominant religion [20]. The purpose of the present study was to cross-culturally translate KIDSCREEN-27 to Bengali language and assess the psychometric capacity of the instrument for assessing HRQoL in adolescents with CP and their peers without disability in Bangladesh.

## Methods

The present study is part of the Bangladesh cerebral palsy health-related quality of life study (Bangladesh CP HRQoL) aimed at determining the HRQoL of adolescents with CP in rural Bangladesh using a population-based sample.

### Participants and study design

Adolescents with CP were identified through the Bangladesh Cerebral Palsy Register (BCPR) using Key Informant Methodology described in Khandaker et al. [9]. BCPR has been operating since January 2015 and is the first population-based register of children and adolescents with CP in an LMIC. The register covers a defined geographical region, the Shahjadpur sub-district of Sirajganj district, in the northern part of Bangladesh and includes 296 villages with a total combined population of 561,076 (child population approx. 226,114) and an estimated 70,998 households [9]. For the present study we attempted to contact all adolescents aged 10 to 18-years registered with BCPR to invite participation. We also requested participation from their primary caregiver classified as a parent, grandparent, other relative or close adult friend who provided the majority of their care and support. Age and sex matched controls were identified using convenience sampling from neighbouring dwellings within the surveillance area.

Informed verbal and written consent was obtained for all individual participants included in the study. Verbal consent was obtained for all minors (i.e. <16y) then written consent was obtained from their parent or legal guardian. In cases where adolescents were unable to provide verbal consent (i.e. due to severe communication impairment or perceived lack of capacity) then consent was only obtained from the parent or legal guardian and data was only collected as proxy data. No data was collected in instances that adolescents indicated objection to participation, even in instances of parental consent. In cases of illiteracy, written consent was obtained by thumbprint. This study has ethical approval from the Bangladesh Medical Research Council (BMRC/NREC/2013–2016/1165) and University of Sydney Human Research Ethics Committee (2016/646). All procedures performed in this study were in accordance with the ethical standards of these institutes and with the 1964 Helsinki declaration and its later amendments or comparable ethical standards.

### Measures

Questionnaires were interviewer administered to adolescents and their primary caregivers. In cases where adolescents appeared unable to understand questions or communicate answers they were excluded from self-reporting and only proxy data was collected.

#### Kidscreen-27

The Bengali version KIDSCREEN-27 questionnaire was administered to all adolescents and their proxies (i.e. cases and controls). The instrument has both self and proxy-report versions and uses Rasch scales to measure participants subjective perception of their wellbeing over the last week using 27 items across five dimensions; physical wellbeing’ (5 items), psychological wellbeing’ (7 items), ‘Autonomy and parents’ (7 items), ‘Peers and social support’ (4 items), and ‘School environment’ (4 items) [21].

We conducted cross-cultural translation of KIDSCREEN-27 following The KIDSCREEN Group Europe [21] translation protocol of forwards and back translation with necessary socio-cultural adaptations. The self and proxy-report English versions of the questionnaire were independently translated by two researchers fluent in both languages but for whom Bengali was their day-to-day language. The translators were given instruction to use natural and acceptable language for the broadest audience and to be simple, clear and concise in their formulations, as well as to focus on conceptual equivalence rather than literal word for word translation. The two translations were compared and assessed for conceptual equivalence, comprehensibility and clarity of speech relative to the original English questionnaires. A reconciled version of each questionnaire was produced as a derision of the two translations.

The reconciled forward translation was back translated by a researcher fluent in both languages but for whom English was their day-to-day language. The same instructions for translation were given to the English translator. The back translation was then compared item-by-item with the original English questionnaires to develop the final forward translation document. Conceptual discrepancies between the translations were resolved. Pre-testing of the final versions (self and proxy questionnaires) was undertaken with eight adolescents and their primary caregivers; translations were determined to be comprehensible and culturally satisfactory and mode of delivery was acceptable. The KIDSCREEN Group Europe provided official approval of the final Bengali version questionnaire. Translation matrix is available in the Additional file 1. The translated questionnaire is available on request from KIDSCREEN.

#### Bangladesh cerebral palsy register (BCPR)

Sociodemographic information and clinical characteristics of adolescents were extracted from the BCPR database including age, sex, body mass index (BMI), gross motor function and classification scale (GMFCS), impairments (in addition to CP), level of education and monthly family income [9].

#### Mental health status

Mental health status was assessed using the Bengali version Strengths and Difficulties Questionnaire (SDQ) [22]. SDQ assesses emotional symptoms, conduct problems, hyperactivity/ inattention, peer relationship problems, and pro-social behavior of people aged 3 to 16 years.

### Statistical analysis

KIDSCREEN-27 scores were converted to T-values (standardised mean = 50, SD = 10) and mean dimension scores calculated by averaging the items in each dimension. ‘Total score’ was calculated as an average of each dimension, dimensions with missing scores were weighted by dividing responses by total number of participants [21]. Data was assessed for normality using Shapiro-Wilk and visual inspection of residual plots. Internal consistency was calculated using Cronbach’s α. This coefficient has a value from 0 to 1; ≥0.70 was considered to indicate high reliability of the instrument for use in group comparison and ≥ 0.90 indicated high reliability for individual patient analysis [23, 24].

Content validity was assessed using confirmatory factor analysis (CFA) to confirm if the underlying dimensions of the translated questionnaire matched the original. Model fit was considered as acceptable if chi-square statistic was *p* > 0.05 and root mean squared error of approximation (RMSEA) was ≤0.08, comparative fit index (CFI) was ≥0.90 and Tucker-Lewis Index (TLI) was ≥0.90 [25]. Exploratory factor analysis (EFA) was undertaken in cases that CFA determined a poor model fit [26]. We conducted principal component analysis with Varimax rotation; factors were disregarded according to visual inspection of Scree plot and if eigenvalue was < 1.0. Forced extraction was conducted to achieve most interpretable solution [26].

Construct validity was determined using the known group’s method [3]; we assessed mean differences in KIDSCREEN-27 outcomes according to; adolescents (a) with CP and (b) without CP [27, 28]; and adolescents with (a) ‘unlikely’, (b) ‘possible’ and (c) ‘probable’ mental health problems using SDQ [17]. Magnitudes of difference between groups in each category were determined by effect size classified as small (≤0.49), medium (0.50 to 0.79), and large (≥0.80) [29]. Concordance between self-report and proxy-report was assessed with intra class correlation (ICC) and comparison of group means (paired samples t-test / Wilcoxon signed rank test pending assumptions of normality). ICC < 0.4 was considered to indicate poor to fair agreement, 0.50 to 0.69 moderate agreement, 0.70 to 0.79 good agreement, > 0.80 excellent agreement [30]. All statistical analysis was conducted using SPSS version 24 (IBM Armonk, NY, USA). A *p* value of < 0.05 was considered significant.

## Results

### Participant characteristics

In total, 64 adolescents with CP, 173 age and sex matched controls and 327 proxies (primary caregivers/ adult guardians) (*n* = 564) participated in this study. Participation rate of adolescents with CP was 80.2% (*n* = 154/192, mean age 15y 1mo, SD 1y 8mo, range 10 to 18y, female *n* = 48, 31.2%). Reasons for non-participation included being unwilling to participate (*n* = 11); no longer living in the surveillance area (*n* = 7); not able to be retraced (*n* = 17); and having deceased (*n* = 3). Adolescents with CP were matched to controls by age and sex (*p* < 0.05). Controls were 173 peers without disability (mean age 14y 9mo, SD 1y 7mo, range 10 to 18y, female *n* = 55, 31.8%). 64 (42%) adolescents with CP provided self-reported HRQoL as did 100% of controls. Primary caregivers provided proxy-reports for all cases and controls. Proxy reporters were mothers (cases *n* = 118; controls *n* = 119), fathers (*n* = 21; *n* = 7) and other primary caregivers (*n* = 15; *n* = 47).

### Feasibility

Missing values, shown in Table 1, were nil for all dimensions except ‘school environment’ (missing cases 48.4 to 74.7% and controls 11.0 to 12.1%). Sub-group analysis revealed missing scores corresponded approximately to rates of non-school attendance (cases self-report 48.4%, cases proxy-report 74.7%, control self- and proxy-report 3.47%).

**Table 1:** Missing scores, floor and ceiling effect and internal consistency (Cronbach’s α), of the Bengali version KIDSCREEN-27

Instrument dimension	Adolescent with CP						Controls				
	Items	*n*	Missing scores *n*(%)	Floor effect *n*(%)	Ceiling effect *n*(%)	Cronbach’s α	*n*	Missing scores *n*(%)	Floor effect *n*(%)	Ceiling effect *n*(%)	Cronbach’s α
SELF-REPORT											
Total score	27	*64*	31 (48.4)	0 (0.0)	0 (0.0)	0.88 ^a^	*173*	21 (12.1)	0 (0.0)	0 (0.0)	0.88 ^c^
Physical wellbeing	5	*64*	0 (0.0)	0 (0.0)	1 (1.6)	0.74	*173*	0 (0.0)	0 (0.0)	0 (0.0)	0.75
Psychological wellbeing	7	*64*	0 (0.0)	0 (0.0)	0 (0.0)	0.75	*173*	0 (0.0)	0 (0.0)	0 (0.0)	0.76
Autonomy and parents	7	*64*	0 (0.0)	0 (0.0)	1 (1.6)	0.73	*173*	0 (0.0)	0 (0.0)	6 (3.5)	0.76
Peers and social support	4	*64*	0 (0.0)	1 (1.6)	1 (1.6)	0.71	*173*	0 (0.0)	2 (1.2)	4 (2.3)	0.67
School environment	4	*64*	31 (48.4)	0 (0.0)	3 (4.7)	0.86 ^a^	*173*	21 (12.1)	0 (0.0)	18 (10.4)	0.69 ^c^
PROXY-REPORT											
Total score	27	*154*	115 (74.7)	0 (0.0)	0 (0.0)	0.89 ^b^	*173*	19 (11.0)	0 (0.0)	0 (0.0)	0.91 ^d^
Physical wellbeing	5	*154*	0 (0.0)	0 (0.0)	0 (0.0)	0.81	*173*	0 (0.0)	0 (0.0)	5 (2.9)	0.91
Psychological wellbeing	7	*154*	0 (0.0)	1 (0.7)	0 (0.0)	0.81	*173*	0 (0.0)	0 (0.0)	0 (0.0)	0.79
Autonomy and parents	7	*154*	0 (0.0)	2 (1.3)	0 (0.0)	0.73	*173*	0 (0.0)	0 (0.0)	3 (1.7)	0.78
Peers and social support	4	*154*	0 (0.0)	36 (23.4)	0 (0.0)	0.83	*173*	0 (0.0)	2 (1.2)	4 (2.3)	0.81
School environment	4	*154*	115 (74.7)	0 (0.0)	1 (0.7)	0.82 ^b^	*173*	19 (11.0)	0 (0.0)	13 (7.5)	0.76 ^d^

^a^ *n* = 33, ^b^ *n = 39;* ^c^ *n* = 152; ^d^ *n* = 154

### Floor and ceiling effects

Floor and ceiling effects, shown in Table 1, were observed as nil or weak on most dimensions (self- and proxy-report ≤4.7%). Moderate ceiling effect was observed in controls for ‘school environment’ (7.5 to 10.4%). Strongest floor effect was observed in adolescents with CP proxy-report for ‘peers and social support’ (23.4%). Sub-group analysis by BMI, school attendance, and monthly family income revealed no strong floor or ceiling effects (self and proxy-report <15%) confirming good sensitivity.

### Internal consistency

Internal consistency, shown in Table 1, was excellent for cases in both self- and proxy-report questionnaires (Cronbach’s α self-report 0.71 to 0.88, proxy-report 0.73 to 0.89). Internal consistency was good to excellent for controls (Cronbach’s α self-report 0.67 to 0.88, proxy-report 0.76 to 0.91).

### Validity

#### Content validity

CFA on the self-report questionnaire showed the original five-factor model to be a poor fit (Chi-square = 747.62, *df* = 314, *p* < 0.001; RMSEA = 0.09 (95% CI 0.08 to 0.09); CFI = 0.72; TLI = 0.68). EFA was then conducted as sampling adequacy was acceptable (Bartlett = < 0.001, KMO = 0.802). EFA produced a seven-factor solution with eigenvalues greater than one and explained 59.9% of the total variance. Forced Factor extraction resulted in a final six-factor solution that explained 56.05% of the total variance. This solution, see Table 2, was most interpretable and corresponded within one to three factors with the original KIDSCREEN-27 dimensions. Specifically, the original ‘physical wellbeing’ dimension fit within one factor. ‘Psychological wellbeing’ fit within two factors; the same factor as ‘physical wellbeing’ for items 6, 7, 8 and 12 and a separate factor for items 9, 10 and 11. Autonomy and parents split across three factors; items 13, 14, 15, 17 as one factor, item 16 as another factor alongside items from the original ‘school environment’ dimension; and items 18 and 19 as alongside item 23 from the original ‘peers and social support’ dimension. The remaining items from ‘peers and social support’ fitted within one factor. All factor loadings were ≥ 0.40 except Item 1 and 8.

**Table 2:** Self-report item factor loadings

	Factor 1	Factor 2	Factor 3	Factor 4	Factor 5	Factor 6
Physical wellbeing						
1. In general, how would you rate your health?	0.30					
2. Have you felt fit and well?	0.66					
3. Have you been physical active?	0.68					
4. Have you been able to run well?	0.43					
5. Have you felt full of energy?	0.72					
Psychological wellbeing						
6. Have you felt that life was enjoyable?	0.56					
7. Have you been in a good mood?	0.49					
8. Have you had fun?	0.35					
9. Have you felt sad?					0.74	
10. Have you felt so bad that you didn’t want to do anything?					0.76	
11. Have you felt lonely?					0.58	
12. Have you been happy with the way you are?	0.49					
Autonomy and Parents						
13. Have you had enough time for yourself?				0.52		
14. Have you been able to do the things that you want to do in your free time?				0.49		
15. Have you felt that your parent(s) had enough time for you?				0.81		
16. Have you felt that your parent(s) treated you fairly?			0.62			
17. Have you been able to talk to your parents(s) when you wanted to?				0.65		
18. Have you had enough money to do the same things as your friends?		0.79				
19. Have you felt that you had enough money for your expenses?		0.78				
Peers and social support						
20. Have you spent time with your friends?						0.81
21. Have you had fun with your friends?						0.75
22. Have you and your friends helped each other?						0.46
23. Have you been able to rely on your friends?		0.57				
School environment						
24. Have you been happy at school?			0.65			
25. Have you got on well at school?			0.74			
26. Have you been able to pay attention?			0.46			
27. Have you got along well with your teachers?			0.70			
Eigenvalue	6.89	1.98	1.89	1.76	1.49	1.14
Percent variance	25.48%	7.33%	6.99%	6.52%	5.52%	4.21%

Extraction method: Principal component analysis; Rotation method: Varimax with Kaiser Normalization

CFA on the proxy-report questionnaire showed the original five-factor model to be a poor fit (Chi-square = 845.27, *df* = 314, *p* < 0.001; RMSEA = 0.10 (95% CI 0.09 to 0.10); CFI = 0.75; TLI = 0.72). EFA was then conducted as sampling adequacy was acceptable (Bartlett = < 0.001, KMO = 0.842). A seven-factor model with eigenvalues greater than one was produced explaining 67.0% of the total variance. The seven-factor solution, see Table 3, corresponded within one to two factors with the original KIDSCREEN-27 dimensions. The original ‘Physical wellbeing’ dimension fit within one factor with exception of item 2 which fit with items 6, 7 and 8 from the original ‘psychological wellbeing’ dimension. The remaining items 9, 10, 11 and 12 from the original ‘psychological wellbeing’ dimension fit within one factor. ‘Autonomy and parents’ split across two factors; items 13, 14, 15, 16 and 17 as one factor; and items 18 and 19 as another. ‘Peers and social support’ and ‘school environment’ each fit within one factor each. All factor loadings were ≥ 0.40.

**Table 3:** Proxy-report item factor loadings

KIDSCREEN-27 Dimensions and Items	Factor 1	Factor 2	Factor 3	Factor 4	Factor 5	Factor 6	Factor 7
Physical wellbeing							
1. In general, how would your child rate her/ his health?		−0.43					
2. Has your child felt fit and well?				0.67			
3. Has your child been physical active?		0.72					
4. Has your child been able to run well?		0.75					
5. Has your child felt full of energy?		0.69					
Psychological wellbeing							
6. Has your child felt that life was enjoyable?				0.67			
7. Has your child been in a good mood?				0.66			
8. Has your child had fun?				0.51			
8. Has your child felt sad?						0.78	
10. Has your child felt so bad that he/she didn’t want to do anything?						0.81	
11. Has your child felt lonely?						0.60	
12. Has your child been happy with the way he/ she is?						0.46	
Autonomy and Parents							
13. Has your child had enough time for him/herself?	0.75						
14. Has your child been able to do the things that he/she wants to do in his/her free time?	0.64						
15. Has your child felt that his/her parent(s) had enough time for him/her?	0.85						
16. Has your child felt that his/her parent(s) treated him/her fairly?	0.45						
17. Has your child been able to talk to his/her parents(s) when he/she wanted to?	0.68						
18. Has your child had enough money to do the same things as his/her friends?							0.86
19. Has your child felt that he/she had enough money for his/her expenses?							0.86
Peers and social support							
20. Has your child spent time with his/her friends?			0.78				
21. Has your child had fun with his/her friends?			0.79				
22. Have your child and his/her friends helped each other?			0.70				
23. Has your child been able to rely on his/her friends?			0.71				
School environment							
24. Has your child been happy at school?					0.61		
25. Has your child got on well at school?					0.61		
26. Has your child been able to pay attention?					0.74		
27. Has your child got along well with his/her teachers?					0.76		
Eigenvalue	8.57	2.67	2.09	1.55	1.30	1.19	1.02
Percent variance	31.74%	8.76%	7.73%	5.74%	4.83%	4.41%	3.79%

Extraction method: Principal component analysis; Rotation method: Varimax with Kaiser Normalization

#### Construct validity

The differences between groups according to presence of CP (i.e. case or control) and mental health status (using SDQ total difficulties ‘unlikely’, ‘possible’ or ‘probable’) are shown in Table 4. Significant differences were observed between groups with and without CP (mean difference 5.5 (95% CI 2.1 to 9.0) to 16.7 (95% CI 14.5 to 18.8), *p* < 0.05, ES ≤0.50). The questionnaires discriminated between SDQ groups for most but not all dimensions (mean difference 5.0 (95% CI 0.5 to 9.4) to 15.5 (95% CI 12.1 to 18.9), ES < 0.40 *p* < 0.05). No difference was observed when analysed by socioeconomic status measured as monthly family income (*p* > 0.05).

**Table 4:** Mean difference in KIDSCREEN-27 proxy scores between adolescents with and without CP and according to mental health status (SDQ)

Instrument dimension	CP to control mean difference (95% CI)	Effect size (ES)	SDQ ‘unlikely’ to ‘possible’ mean difference (95% CI)	SDQ ‘unlikely’ to ‘probable’ mean difference (95% CI)	SDQ ‘possible’ to ‘probable’ mean difference (95% CI)	Effect size (ES)
Total score	14.8 (13.1 to 16.4) **	0.50	7.6 (3.6 to 11.6) *	12.6 (10.2 to 15.0) *	5.0 (0.8 to 9.2) *	0.31
Physical wellbeing	16.7 (14.5 to 18.8) **	0.42	8.8 (4.5 to 13.1) *	13.8 (10.5 to 17.0) *	5.0 (0.5 to 9.4) *	0.24
Psychological wellbeing	16.3 (13.9 to 18.8) **	0.35	10.2 (5.7 to 14.7)*	15.5 (12.1 to 18.9) *	5.3 (0.6 to 10.0) *	0.27
Autonomy and parents	8.0 (5.8 to 10.1) **	0.14	5.4 (1.5 to 9.3) *	6.7 (3.8 to 9.6) *	1.3(−2.7 to 5.4)	0.09
Peers and social support	12.3 (9.8 to 14.8) **	0.23	5.5 (0.8 to 10.3) *	11.4 (7.9 to 14.8) *	5.8 (0.6 to 11.1) *	0.16
School environment ^b^	5.5 (2.1 to 9.0) **	0.05	1.0(−4.1 to 6.1)	6.9 (2.8 to 11.0) *	5.9 (0.0 to 11.1)	0.08

** significant at 0.01 level; * significant at the 0.05 level

^a^Adolescents with CP *n* = 39; Controls *n* = 154

### Concordance between self and proxy-report

ICC, Table 5, was moderate to excellent for all dimensions for both cases and controls (0.5 to 0.8). Proxies estimated poorer HRQoL on all dimensions of which mean difference was significant for three dimensions in cases (2.2 to 3.8); and four dimensions for controls (1.4 to 3.1).

**Table 5:** ICC and mean difference between self and proxy-reported KIDSCREEN-27

Instrument Dimension	Adolescents with CP (*n* = 64)			Controls (*n* = 173)		
	ICC (95% CI)	Mean difference (95% CI)	*p*-value	ICC (95% CI)	Mean difference (95% CI)	*p*-value
Total score	0.6 (0.4 to 0.8)	2.19 (0.7 to 3.7)	**0.010**	0.8 (0.7 to 0.9)	2.2 (1.2 to 3.1)	**<0.001**
Physical wellbeing	0.7 (0.5 to 0.8)	2.42 (−0.4 to 5.2)	0.132	0.7 (0.7 to 0.8)	4.7 (3.0 to 6.5)	**<0.001**
Psychological wellbeing	0.5 (0.2 to 0.7)	3.82 (1.2 to 6.4)	**0.002**	0.7 (0.6 to 0.8)	0.2 (−1.4 to 1.8)	0.597
Autonomy & parents	0.7 (0.5 to 0.8)	2.65 (0.6 to 4.7)	**0.015**	0.8 (0.7 to 0.8)	3.1 (1.9 to 4.3)	**<0.001**
Peers and social support	0.5 (0.1 to 0.7)	1.73 (−0.8 to 4.3)	0.339	0.6 (0.5 to 0.7)	1.4 (−0.1 to 2.9)	**0.094**
School environment ^a^	0.6 (0.2 to 0.8)	3.40(−0.7 to 7.5)	0.114	0.6 (0.5 to 0.7)	2.4 (0.8 to 4.0)	**0.002**

^a^Adolescents with CP *n* = 33; Controls *n* = 152

## Discussion

To the best of our knowledge, this is the first validation study of KIDSCREEN-27 in Bangladesh, and one of a selected few conducted in an LMIC or predominately Islamic country. Our study demonstrated that the Bengali version KIDSCREEN-27 self and proxy-report questionnaires have overall good psychometric properties and are reliable and valid measures for use in Bangladesh, including with adolescents with CP and age and sex matched peers without disability.

We used a population-based sample involving case-control comparison of adolescents with CP and age and sex matched peers without disability. Moreover, in accordance with good practice on the conduct of HRQoL research we attained language, operational and scale equivalence as part of our translation and adaptation procedure [23, 31, 32]. Multistage forward and back translation with pilot testing ensured that we achieved appropriate language and socio-cultural adaptations; we interviewer administered the questionnaires to account for low levels of literacy within our target population; and we confirmed acceptability of the instrument administration time frame and conceptual understanding of the measurement scale during pilot testing.

We collected self-reported data from adolescents in all instances possible. We also collected proxy data to enable inclusion of adolescence with severe cognitive or communication impairments. To understand the agreement between self and proxy-reported HRQoL we conducted case-wise comparison. We found similar agreement for cases and controls and, in accordance with other research, dimensions with more observable components, for example ‘physical wellbeing’ showed stronger agreement and dimensions with less observable components for example ‘peers and social support’ had weaker agreement [33].

Both self and proxy-report versions of the Bengali KIDSCREEN-27 questionnaire for cases and controls reported psychometric properties comparable to instrument norms [17]. For example, internal consistency of the original European KIDSCREEN-27 was 0.78 to 0.81, and for the Iranian (Persian) version (the other Islamic LMIC for which KIDSCREEN-27 has been translated) was 0.73 to 0.85 [38]. Our results were similarly good; both questionnaires approached or exceeded scale cut offs (Cronbach’s α < 0.70) for group comparison; although majority of dimensions were Cronbach’s α < 0.90 and so further testing should be undertaken before use in individual patient analysis. Exceptions in psychometric comparison were high proportions of missing scores in ‘school environment’ and strong floor effect in ‘peers and social support’ for adolescents with CP. These two results may hint that the instrument is not uniquely sensitive to the adolescents with CP in our sample. Despite national ‘education for all’ policies non-school attendance for adolescents with disability in Bangladesh is common; as is social isolation due to stigma about disability and lack of infrastructure i.e. wheelchairs, footpaths, ramps [34]. Moreover, physical impairment was more severe in our sample than comparable research from HICs and may account for the observed findings and justify future research with instrument adaption using a lower sensitivity threshold.

KIDSCREEN-27 discriminated between groups with known differences in HRQoL including adolescents with CP compared to controls and between adolescents with ‘unlikely’, ‘possible’ or ‘probable’ mental health status using SDQ. Our findings confirm that of other validation studies [35, 36] although our effect sizes were small, possibly due to small sample size.We did not find difference in outcomes according to monthly family income although this may be due to homogeneity in our sample and use of different measure of socioeconomic status. The previous studies used a more sensitive measure of socioeconomic status, ‘Family Affluence Scale’. Administration of the Bengali KIDSCREEN-27 questionnaire amongst adolescents with CP in other geographic regions of Bangladesh as well as in West Bengal state of India, where Bengali is the main language spoken, is recommended. This study has generated the first normative data for adolescents without disability in Bangladesh, although larger studies are required to confirm our findings.

Confirmatory factor analysis found that the underlying five-factor structures of the original questionnaires were a poor fit. Results suggested that a six and seven factor solution for the self and proxy-report questionnaires, respectively, be used. Several studies including Ng, Burnett [36] and Shannon, Breslin [37] have reported variation in the dimension structures of KIDSCREEN-27 when translated for use in other contexts involving the addition of two dimensions ‘moods and emotions’ and ‘financial resources’. Our findings lean towards the seven-factor model described in Ng, Burnett [36] however further research with larger sample sizes is necessary to confirm our findings prior to development of a new dimension structure for the Bengali version KIDSCREEN-27 questionnaire.

Overall, our psychometric testing has indicated that the Bengali version KIDSCREEN-27 questionnaire performs well however there are limitations including that it was outside the scope of this study to determine how HRQoL was conceptualised amongst our target population. Conceptualisation of HRQoL can be culturally specific, impacted by linguistic, religious and cultural variations. Strength of KIDSCREEN is that the development methodology of the original questionnaires was multinational, and intended to reflect internationally defined multidimensional theoretical constructs of HRQoL [3]. Further investigation of conceptualisation of HRQoL in Bangladesh will strengthen future research as would study on measurement equivalence to determine cross-cultural comparability of KIDSCREEN-27 results between Bangladesh and other contexts in which KIDSCREEN-27 has been tested [17, 18]. Moreover, due to resource restraints we do not measure test-retest reliability, structural validity, or sensitivity to change [4, 32]. Further testing with larger sample sizes in other geographical and sociodemographic regions of Bangladesh and West Bengal state of India is recommended to confirm our findings.

## Conclusion

The Bengali version KIDSCREEN-27 self and proxy-report questionnaires underwent rigorous procedure for cross-cultural translation and reported good psychometric properties indicating suitability for use in group comparison with adolescents with CP and controls without disability in Bangladesh. ‘School environment’ required further investigation as to its applicability to adolescents with CP due to high levels of non-school attendance within our sample and children with disability in Bangladesh. Similarly, the capacity of ‘peers and social support’ to capture the experiences of adolescents with CP requires further investigation. Overall KIDSCREEN-27 is a valid and reliable measure for assessing HRQoL in adolescents in Bangladesh.

## Additional file

Additional file 1: KIDSCREEN 27 - National Translation Procedure. (XLSX 37 kb)

## Abbreviations

BMI: Body mass index
CP: Cerebral palsy
CPQoL-Teens: Cerebral palsy quality of life teens questionnaire
ES: Effect size
GMFCS: Gross motor function classification system
HIC: High-income country
HRQoL: Health-related quality of life
LMIC: Low and middle-income country
SD: Standard deviation
